# RA-risk synovium exhibits DNA damage coupled with impaired DNA repair in fibroblasts

**DOI:** 10.1136/rmdopen-2025-005774

**Published:** 2026-01-22

**Authors:** Aoife M O’Byrne, Tineke A de Jong, Johanna F Semmelink, Przemek M Krawczyk, Ron A Hoebe, Marleen van de Sande, Lisa van Baarsen

**Affiliations:** 1 Department of Rheumatology & Clinical Immunology, Amsterdam UMC Locatie AMC, Amsterdam, The Netherlands; 2 Laboratory for Experimental Immunology, Amsterdam UMC Locatie AMC, Amsterdam, The Netherlands; 3 Department of Rheumatology and Clinical Immunology, University of Amsterdam, Amsterdam, The Netherlands; 4 Department of Rheumatology and Clinical Immunology, Amsterdam UMC Locatie AMC, Amsterdam, The Netherlands; 5 Department of Medical Biology, Amsterdam UMC Locatie AMC, Amsterdam, The Netherlands; 6 Amsterdam Rheumatology and Immunology Center, Amsterdam, The Netherlands

**Keywords:** Arthritis, Rheumatoid, Fibroblasts, T-Lymphocytes, Autoimmunity

## Abstract

**Objectives:**

Understanding the molecular changes in the preclinical synovium is crucial for identifying factors that drive arthritis development. Persistent DNA damage in tissues is known to drive a senescent microenvironment, genomic instability and ultimately chronic inflammation. Here, we determined cellular DNA damage and repair capacity within synovial tissue from rheumatoid arthritis (RA) patients and individuals at risk of developing RA.

**Methods:**

We investigated the presence of senescence-associated DNA damage in synovial biopsies and synovial fibroblasts obtained during different phases of RA. Histone 2A is phosphorylated (γH2AX) at the site of a double-stranded DNA break where DNA repair proteins are recruited and is therefore a proxy measurement for DNA damage. In this study, we employed immunofluorescence staining for γH2AX on synovial tissue sections and cultured synovial fibroblasts alongside quantitative PCR for a panel of DNA repair proteins.

**Results:**

We demonstrated the presence of DNA damage in both synovial fibroblasts and T cells during the preclinical, RA-risk phase of disease. Furthermore, cultured synovial fibroblasts from RA-risk individuals and RA patients exhibited increased DNA damage and a reduced capacity for DNA repair compared with synovial fibroblasts from control individuals. Finally, treatment with senolytic drugs partially restored the DNA damage repair capacity in RA and RA-risk synovial fibroblasts in vitro.

**Conclusions:**

Our findings reveal persistent DNA damage in the preclinical phase of RA in both synovial tissue and fibroblasts, suggesting a role in disease progression. The partial restoration of DNA repair in synovial fibroblasts by senolytic treatment highlights its potential therapeutic target for preventative therapy in RA-risk individuals.

**WHAT IS ALREADY KNOWN ON THIS TOPIC**

- Synovial biopsies of rheumatoid arthritis (RA)-risk individuals without subclinical inflammation are characterised by a low T cell infiltrate, which is modestly associated with the development of arthritis, despite the absence of clear signs of synovial inflammation. DNA damage is a hallmark of senescence, which has been observed in immune cells, specifically T cells, from the peripheral blood of RA patients and within the synovium of RA patients with established disease.

**WHAT THIS STUDY ADDS**

- This study is the first to demonstrate, to our knowledge, senescence-associated DNA damage in both T cells and fibroblasts within the synovium of RA-risk individuals. Our findings reveal increased DNA damage in RA and RA-risk synovial fibroblasts, accompanied by a reduced DNA repair capacity, which can be partially restored by senolytic treatment targeting senescent cells. This advances the understanding of the dysfunctional state of synovial fibroblasts in the early stages of RA development, potentially contributing to disease progression.

**HOW THIS STUDY MIGHT AFFECT RESEARCH, PRACTICE OR POLICY**

- These findings underscore that cellular processes commonly studied in oncology and the biology of ageing, such as DNA damage and senescence, may have direct relevance for rheumatology by shaping the preclinical phase of RA. It provides evidence for targeting senescent cells within synovium as a potential therapeutic approach, opening new avenues for intervention studies in RA-risk individuals.

## Introduction

 Rheumatoid arthritis (RA) is a chronic inflammatory autoimmune disease of unknown aetiology which preferentially affects joints leading to tissue destruction and pannus formation.^1 2^ Seropositive RA is preceded by a preclinical at-risk phase where there are clear signs of a break in immunological tolerance. This is indicated by the presence of autoantibodies against citrullinated peptides (ACPA) and rheumatoid factor (RF), which can be accompanied with arthralgia.^3 4^ Of all those at risk of developing RA (RA-risk), only 30% of individuals^4^,^6^ go on to develop the disease within 2 years suggesting there are additional (environmental) factors^5^ and genetic predispositions^7^,^9^ that may drive progression to clinical arthritis. Longitudinal prospective cohort studies of RA-risk individuals give us the opportunity to study disease pathogenesis and initiate preventative intervention studies. Retrospective analysis of such cohorts allows us to identify pre-RA individuals within the prospectively followed cohort of RA-risk individuals. This enables us to compare samples of RA-risk individuals that will develop arthritis termed pre-RA to those that are still considered RA-risk but may never develop disease. Differentiating RA-risk from pre-RA individuals in this preclinical phase remains a strong research focus given the therapeutic benefits of early intervention and prevention.

The immune landscape of the synovial joint has been extensively explored in patients with RA, demonstrating distinct immunological subtypes based on levels of immune cell infiltrate,^10^ which is further supported by single-cell RNA sequencing studies.^11^,^14^ Numerous immune profiling studies described the immunological players that may drive chronic inflammation within the joint^1^,^3^ with both polyfunctional T cells^15^ and maladaptive fibroblasts (FLS)^16^ being implicated in active disease. Several synovial tissue studies have highlighted that when accounting for disease activity and treatment, the cellular infiltrate and inflammatory cytokine profile within the joint is comparable between early and established disease.^17^,^21^ This knowledge emphasises the importance of studying the preclinical disease phase as the initiating events of synovitis are still unknown. Our previous studies exploring the synovial landscape in prospectively followed RA-risk individuals highlight a subtle infiltration of T cells^22^ preceding arthritis development and the absence of subclinical inflammation.^23^ Transcriptional profiling highlights molecular changes in key genes associated with resident synovial cells such as FLS.^24^ Despite our growing knowledge of the RA synovial tissue microenvironment, the key drivers of these aberrant immune cell changes within synovial joints have yet to be fully elucidated.

This aberrant synovial tissue microenvironment preceding RA onset in the absence of inflammation may allude to intrinsic maladaptive tissue resident cells within the joint. Growing knowledge indicates that RA may be caused by premature ageing of the immune system. A number of the physiological hallmarks of an ageing immune system have been identified in RA,^25^ including telomere shortening,^26^,^28^ mitochondrial dysfunction,^29 30^ cellular senescence, aberrant immune cell signalling known as inflammageing and genomic instability.^31^ DNA damage is a process that occurs in all cells, increases with age and is tightly controlled by DNA repair mechanisms.^32^ Accumulation and persistence of DNA damage contribute to genomic instability and a senescent microenvironment.^33^ Increased DNA damage and dysfunctional DNA repair have been observed in peripheral blood T cells from RA patients compared with healthy controls.^34^,^36^ This combined with known telomere shortening in peripheral blood RA T cells^26^ suggests genomic instability within the periphery. Early synovial tissue studies highlight increased DNA fragmentation^37^ and p53 expression by synovial FLS^38^ within RA synovial tissue lining, indicative of genomic instability. p53 is a well-established central transcriptional regulator of the DNA damage response,^39^,^41^ which is overexpressed in both early and established RA synovium.^42^ Together with evidence that genotoxic stress can induce DNA damage, leading to p53 mutations in FLS,^43^ this supports a potential role for p53 dysregulation in driving synovial genomic instability. Current evidence has yet to determine whether this genomic instability is present before disease onset or is a product of the chronic inflammatory environment fostered in established disease. In this study, we sought to determine whether senescence-associated DNA damage is present in synovial cells during the preclinical RA-risk phase. We employed our previously studied synovial RA-risk cohort where we were able to retrospectively select those RA-risk individuals who went on to develop arthritis (pre-RA) and those who remained at risk.^24^ Subsequently, we determined the level of DNA damage and DNA repair capacity by examining synovial tissue and cultured FLS for the presence of phosphorylated histone 2AX (γH2AX) by immunofluorescence. Histone 2AX is phosphorylated at serine 139 on double-stranded DNA breaks^44^ where it signals for the recruitment of DNA repair machinery and therefore acts as a widely accepted proxy measurement for DNA damage.^45^,^50^

## Methods

### Study cohorts

Individuals with arthralgia and/or a family history of RA who were positive for IgM-RF and/or ACPAs but without any current or previous evidence of arthritis on examination or a diagnosis of RA underwent miniarthroscopic synovial tissue sampling of a knee joint at baseline.^22 51^ These individuals are considered to be at risk of developing RA (RA-risk individuals, defined as phase c+d, according to EULAR recommendations).^52^ Study subjects were recruited either at the AMC outpatient clinic, via referral from the rheumatology outpatient clinic of Reade, Amsterdam, or by autoantibody testing of family members of RA patients. For comparison, synovial tissues of RA (ACR/EULAR 2010 criteria^52^), osteoarthritis (OA) and gout patients were collected during joint replacement surgery or mini-arthroscopy of a knee joint^2^ and control synovial tissue from seronegative individuals during exploratory orthopaedic knee surgery due to joint injury. These control individuals had no history of autoimmunity or inflammatory disease and were not at present or previously taking any disease-modifying antirheumatic drugs or biologics. The study was performed according to the principles of the Declaration of Helsinki,^53^ approved by the Institutional Review Board of the Amsterdam UMC, location AMC, Netherlands and all study subjects gave their written informed consent.^22^

### Synovial tissue biopsy sampling and FLS culture

Study subjects underwent miniarthroscopic synovial biopsy sampling of a knee joint.^22^ For the synovial tissue cohort, per individual, for both microscopy and RNA isolation, between six and eight synovial tissue biopsies were collected and immediately snap frozen for qPCR or first embedded in Tissue-Tek OCT (Labtech) blocks for immunofluorescence microscopy studies. Synovial tissue sections (5 um) were cut and mounted on StarFrost adhesive glass slides (Knittel Glass, StatLab) before being sealed and stored at −80°C until required. For the FLS cohort, synovial biopsies were processed as previously described.^54^ FLS were cultured in complete cell culture media consisting of Dulbecco’s Modified Eagle Medium, low glucose (Gibco, Bleiswijk, The Netherlands) supplemented with 1% penicillin/streptomycin (10′000 U/mL, Gibco), 10 mM 4-(2-hydroxyethyl)−1-piperazineethanesulfonic acid buffer (Gibco) and 10% fetal bovine serum (Biowest, Nuaillé, France). For all experiments, FLS were used between passages 3 and 5.

### Immunofluorescence

Synovial tissue sections were thawed at room temperature for 1 hour followed by fixation with 4% paraformaldehyde (PFA) in PBS for 10 min. Subsequently, sections were washed with PBS before incubation with 0.2% Triton x100 at room temperature for 10 min to allow for permeabilisation. Following further washing with PBS, sections were incubated with PBS containing 1% bovine serum albumin (BSA) for 30 min at room temperature to prevent non-specific binding. Sections were then stained with primary monoclonal antibodies for CD3 (CD3-12, BioRad) and γH2AX (JBW301, Sigma‒Aldrich) in PBS containing 1% BSA. Sections were incubated for 1 hour and then washed with PBS to remove excess antibody. Sections were subsequently stained with goat anti-rat IgG1 and goat mouse IgG1 secondary antibodies, respectively (Invitrogen) and an AlexaFluor-488 conjugated vimentin antibody (O91D3, Biolegend). All antibodies were suspended in PBS 1% BSA containing 10% human serum and incubated for 1 hour in the dark. On incubation, sections were washed with PBS and stained for DAPI (ThermoFisher Scientific) for 5 min in the dark at room temperature. Sections were washed with PBS and tap water, dried and mounted using VectaMount Mounting Medium (Vector Laboratories). Sections were kept at 4°C until image acquisition.

FLS were seeded in triplicate at a density of 25 000 cells on sterilised 12 mm round coverslips (Knittel-Glaeser, Bielefeld, Germany) placed in 24-well plates (Corning, Amsterdam, The Netherlands) and cultured for 24 hours at 37 °C/ 5% CO_2_. Subsequently, cells were washed with warm PBS and fixed with 4% PFA for 10 min at room temperature (RT). After washing with PBS, cells were permeabilised for 60 min at RT using PBS containing 1% BSA and 0.1% saponin (Sigma‒Aldrich). Cells were then stained overnight at 4°C with monoclonal mouse IgG anti-human γH2AX (JBW301, Sigma‒Aldrich). The next day, cells were washed with PBS+1% BSA + 0.1% saponin and incubated for 30 min at 4°C with secondary anti-mouse IgG1 AlexaFluor633 (Invitrogen, Landsmeer, the Netherlands). After washing with PBS, coverslips were removed from the wells, left to dry at RT in the dark, and mounted on microscope slides with DAPI Vectashield Hardset mounting media (Vector Laboratories).

### Confocal microscopy and image analysis

Synovial tissue sections were imaged using Mica wide field microscope (Leica Microsystems) at 10x objective and FLS imaged using either the TCS SP8 or DMi8 confocal microscope (Leica Microsystems). Images were subsequently analysed using QuPath V.0.5.0.^55^ Synovial tissue sections were analysed by first outlining the total tissue area per image. Cell segmentation was performed using the StarDist extension^56^ on DAPI-stained nuclei, with segmented regions expanded to approximate the cellular cytoplasm. Marker expression was quantified by average pixel intensity within defined compartments: CD3 across the whole cell, vimentin restricted to the cytoplasm and γH2AX restricted to the nucleus. Pixel intensity thresholds for positivity were optimised and used to train object classifiers for each marker, with additional filtering to exclude non-specific deposits. Data from all nucleated cells within each tissue section were included in downstream quantitative analyses. For FLS analysis, background signal was removed and thresholds set using LAS X software (Leica Microsystems). Images were then analysed in QuPath where nucleated cells were segmented as described above using the StarDist extension.^56^ The number of foci per cell was then determined by the number of γH2AX regions per DAPI region in approximately 50 cells per sample. Graphs representing this quantitative analysis show the average of technical replicates and were created using GraphPad V.10.2.0.

### FLS culture, irradiation and senolytic treatment

For DNA damage repair analysis, FLS were irradiated at 1 Gray using a CellRad+ (Precision X-ray, Madison, Madison). Immunofluorescence staining was performed as described above for γH2AX either directly, 3 hours or 24 hours postirradiation. In some conditions, cultured FLS were pretreated with 0.25 µM dasatinib (MedChemExpress, Monmouth Junction, New Jersey) for 24 hours to eliminate senescent cells. Subsequently, culture flasks were washed and cultured to at least 80% confluence based on the flask containing untreated cells.

### qPCR

Total RNA was extracted using the RNA micro kit (Qiagen, Venlo, The Netherlands) following the manufacturer’s guidelines. cDNA synthesis was then performed with the RevertAid H Minus First Strand cDNA Synthesis Kit (Thermo Fisher Scientific, Landsmeer, The Netherlands). Quantitative PCR was conducted using either TaqMan Universal PCR master mix combined with TaqMan assays for MutL protein homologue 1 (MLH1), MutS homologue 2 (MSH2), postmeiotic segregation increased 2 (PMS2), ERCC excision repair 2 (ERCC2), p53 binding protein 1 (53BP1), RAD51 homologue 1, breast cancer 2 (BRCA2) or the fast SYBR Green PCR master mix (Applied Biosystems, Life Technologies, Zwijndrecht, The Netherlands) with custom-designed primers (Sigma-Aldrich) for the following genes: Lamin B1 (LMNB1), forkhead box O4 (FOXO4), B cell lymphoma 2 like 1 (BCL2L1), galactosidase beta 1 (GLB1), ephrin B1 (EFNB1), CD38, nicotinamide phosphoribosyltransferase (NAMPT), Poly(ADP-ribose) polymerase 1 (PARP1), superoxide dismutase 2 (SOD2), MRE11 homologue A (MRE11A), sirtuin 1 (SIRT1), tumour protein p53 (p53), interleukin 6 (IL6), cyclin dependent kinase inhibitor 1A (CDKN1A), cyclin dependent kinase inhibitor 2A (CDKN2A), high mobility group box 1 (HMGB1), cyclin dependent kinase 4 (CDK4). Primer sequences are detailed in table 1. Detection was performed using the QuantStudio 3 (Applied Biosystems). The expression levels of each target gene were normalised to the geometric mean of two reference genes: *TATA-box binding protein* and *RNA polymerase II subunit G*. An arbitrary calibrator sample was used to adjust for interplate variability. The relative quantity was calculated using the standard curve method for SYBR Green measurements and using the delta delta Ct method for TaqMan measurements.

**Table 1:** qPCR primer sequences

(A) SYBRgreen			
Gene	mRNA transcriptID	Forward sequence	Reverse sequence
TBP	NM_001172085.1	GTGGGGAGCTGTGATGTGAA	TGCTCTGACTTTAGCACCTGT
POLR2G	NM_002696.3	GAGGTCGTGGATGCTGTTGT	TCTCTGAAGGGATGGAATGTCG
PARP1	NM_001618.4	AACCGAAGATTGCTGTGGCA	ACCAAACATGTAGCCTGTCACG
LMNB1	NM_001198557.1	AAATTCTCAGGGAGAGGAGGT	TTGGATGCTCTTGGGGTTC
MRE11a	NM_005590.3	CAGCCTTCCCGAAATGTCAC	GTGCTGGACCACCTTTGATCT
HMGB1	NM_002128.7	CGACTCTGTGCCTCGCTGA	TTAGCAGACATGGTCTTCCACC
FOXO4	NM_001170931.1	GGAAAAGGCCATTGAAAGCG	ATGAACTTGCTGTGCAGGGA
BCL2L1	NM_138578.3	CTGTGCGTGGAAAGCGTAGA	GCTGCTGCATTGTTCCCATAG
GLB1	NM_001079811.2	TTTGCTCTGCGAAACATCATCC	GCTCCCACTGTCTTTAACTTTTCC
EFNB1	NM_004429.5	GGAGGCAGACAACACTGTCA	TCCTGGTTCACAGTCTCATGC
CD38	NM_000072.3	TGGCTTAATGAGACTGGGACC	TCTATCAGGCCAAGGAGGTT
NAMPT	NM_005746.3	TCTGGAAACCCTCTTGACACTG	GTTTCATGCCTTCTACAATCTCTTG
SOD2	NM_001322820.2	GGCCTACGTGAACAACCTGA	TGGGCTGTAACATCTCCCTTG
SIRT1	NM_001314049.1	GAGCAGATTAGTAGGCGGCTT	CTCAGCGCCATGGAAAATGT
p53	NM_001126116.1	CAGTCACAGCACATGACGGA	GCCAGACCATCGCTATCTGAG
IL6	NM_001318095.2	AGGAGACTTGCCTGGTGAAAA	GTTGGGTCAGGGGTGGTTATT
CDKN1A	NM_078467.2	AGACCAGCATGACAGATTTCTACC	GCGGATTAGGGCTTCCTCTT
CDKN2A	NM_000077.4	TCCCTCAGACATCCCCGATT	CCTGTAGGACCTTCGGTGAC
CDK4	NM_000075.4	TACACCCGTGGTTGTTACAC	AACTGGTCGGCTTCAGAGTTT

(B) TaqMan	
Gene	Assay ID
TBP	Hs00427620_m1
POLR2G	Hs00275738_m1
MLH1	Hs00179866_m1
MSH2	Hs00953527_m1
PMS2	Hs00241053_m1
ERCC2	Hs00361161_m1
53BP1	Hs00996827_m1
RAD51	Hs00947967_m1
BRCA2	Hs01037421_m1

### Statistical testing

**Figure 1:**
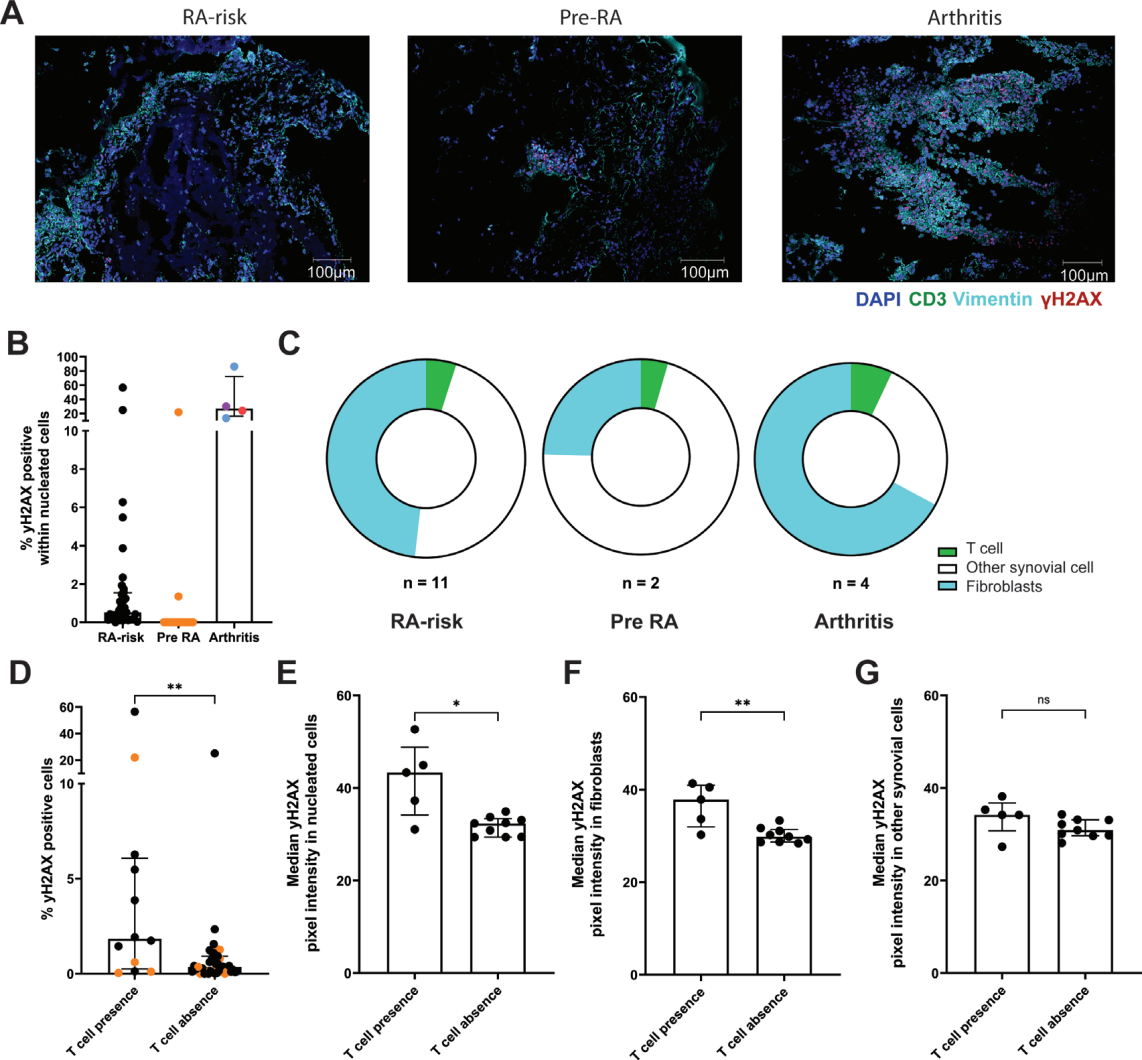
DNA damage in synovial tissue preceding inflammation. Representative images depicting immunofluorescence staining for T cells (green), fibroblasts (cyan), γH2AX positive (pink) and nucleated cells (blue) in synovial tissue (**A**) from RA-risk, pre-RA and arthritis patients with active disease. The presence of γH2AX was determined in all nucleated cells per tissue section detected by the StarDist extension. Graph (**B**) representing the percentage of phosphorylated histone 2AX (γH2AX), indicative of DNA damage, in RA-risk (black circle), pre-RA (orange circle) and arthritis (RA=red, OA=blue and gout=purple circle) synovial tissue sections. Pie charts (**C**) representing the distribution of γH2AX positive cells within T cells (CD3 positive, green), fibroblasts (vimentin positive, blue) and other synovial cells (CD3 and vimentin negative, white) in γH2AX positive synovial tissue sections from RA risk (n=11), pre-RA (n=2) and arthritis (n=4) individuals. Graph (**D**) representing the percentage of γH2AX positive cells within synovial tissue sections positive or negative for T cells, pre-RA samples are represented by orange circles. Graphs from γH2AX positive synovial tissues representing the median γH2AX pixel intensity of total nucleated cells (**E**), vimentin positive fibroblasts (**F**) and other synovial cells (**G**) in T cell positive and negative synovial tissue section. Kruskal-Wallis T test used for statistical analysis of multiple groups. For statistical analysis of T cell presence and absence groups, a Mann-Whitney U test was performed. *p value<0.05, **< 0.01, ns, not significant; RA, rheumatoid arthritis.

GraphPad V.10.2.0 was used to perform statistical testing. A Kruskal-Wallis or two-way Analysis of Variance (ANOVA) followed by Dunn’s or Sidak’s multiple testing was used for unpaired and paired data comparing multiple groups, respectively. For comparison of two independent groups in figure 1, a Mann-Whitney U test was performed.

## Results

Since our previous microscopy studies on the same cohort did not show overt synovial tissue inflammation,^23^ we considered this cohort as at risk without subclinical inflammation. This is important since subclinical inflammation is a risk factor for the development of RA.^57^ Demographics tables for the studied synovial tissue cohort (table 2) and the synovial FLS cohort (table 3) are detailed below. Of the 50 prospectively followed RA-risk individuals included in this synovial tissue cohort, 14 individuals developed arthritis after follow-up (28%). These are referred to as pre-RA samples. Due to limitations in tissue availability as a result of small biopsies of uninflamed tissue, as well as time and labour constraints, FLS could only be expanded from a subset of the synovial tissue cohort. As pre-RA status is only determined retrospectively, unfortunately, none of the donors whose FLS were expanded went on to develop RA in the follow-up period.

**Table 2:** Synovial tissue cohort

Clinical characteristic	RA-risk n=36	Pre-RA n=14
Sex, female, n (%)	25 (69)	9 (64)
Age (years), median (IQR)	49 (35–53)	45 (43–54)
IgM-RF positive/%	53	64
RF level (kU/L), median (IQR)	19.5 (3.5–67.5)	24.0 (5.75–109.0)
ACPA positive	64	79
ACPA level (kAU/L), median (IQR)	106 (3.0–608.3)	352.0 (42.5–1156.0)
IgM-RF and ACPA both pos,%	17	43
ESR (mm/hour), median (IQR)	9.0 (2.0–19.75)	7.5 (5.0–15.0)
CRP (mg/L), median (IQR)	2.0 (1.0–5.375)	3.8 (1.575–10.45)
68 tender joint count, median (IQR)	2.0 (0.0–6.0)	4.5 (0.75–8.50)
Body mass index, median (IQR)	24.62 (22.85–29.21)	27.58 (25.64–29.52)
Visual Analogue Scale of Disease Activity (mm), median (IQR)	29.0 (5.25–66.75)	50.0 (21.50–64.75)
follow-up time months, median (IQR). Total or until arthritis	59 (34–74)	14 (6–34)

ACPA, anti-citrullinated protein antibodies; CRP, C reactive protein; ESR, erythrocyte sedimentation rate; IgM-RF, IgM rheumatoid factor; RA, rheumatoid arthritis.

**Table 3:** Synovial fibroblast (FLS) cohort

	Controls n=6	RA-risk individuals n=6	RA patients n=6
Sex, female, n (%)	1 (16.6)	5 (83.3)	2 (33.3)
Age, years, median (IQR)	54 (27–60)	50 (44–63)	63 (53–66)
IgM-RF positive, n (%)	ND	2 (33.3)	4 (66.6)
ACPA positive, n (%)	ND	4 (66.6)	5 (83.3)
IgM-RF and ACPA both positive, n (%)	ND	0 (0)	3 (50)
ESR (mm/hour), median (IQR)	ND	8.5 (5.0–11.3)	15.5 (6.7–19.7)
CRP (mg/L), median (IQR)	ND	1.5 (0.8–2.0)	3.45 (2.7–4.9)
DAS28, median (IQR)	ND	ND	3.6 (3.2–4.3)
VAS disease activity (mm), median (IQR)	–	19.0 (6.5–41.3)	63.0 (18.5–90.0)
Disease duration (months), median (IQR)	–	–	137 (83–261)
Treatment, n (%)			
NSAID	2 (33.3)	–	3 (50)
Anti-TNF	–	–	3 (50)
Corticosteroids	–	–	1 (16.6)
Rituximab	–	–	1 (16.6)
DMARD	–	–	4 (66.6)

Data are expressed as median (interquartile range) unless otherwise indicated.

ACPA, anti-citrullinated protein antibodies; CRP, C reactive protein; DAS, disease activity score; DMARD, disease modifying anti-rheumatic drug; ESR, erythrocyte sedimentation rate; IgM-RF, IgM rheumatoid factor; ND, not determined; NSAID, non-steroidal anti-inflammatory drug; TNF, tumour necrosis factor; VAS, visual analogue scale.

### DNA damage is present in synovial tissue of individuals at risk of developing arthritis

Immunofluorescence staining was performed on synovial tissue sections from RA-risk individuals to investigate the presence of DNA damage in T cells, FLS or other cells. This staining detected γH2AX, and thus DNA damage, in the synovium of 14 out of the 50 RA-risk individuals and in all included arthritis patients (RA, OA and gout) (figure 1A, online supplemental figure 1). Qualitative assessment revealed that within synovial tissue sections positive for γH2AX, there were also FLS and T cell regions, which showed no γH2AX staining and therefore did not exhibit DNA damage. Subsequent quantitative analysis showed an increased percentage of γH2AX positive cells in synovium of patients with established arthritis compared with RA-risk (p value=0.0143) and pre-RA synovium (p value=0.0019, figure 1B). The percentage of γH2AX positive cells in synovium of RA-risk individuals was highly variable, and positivity did not predict arthritis development (Cox regression analysis p>0.05). Furthermore, the total percentage of γH2AX positive cells did not correlate with any clinical parameters (online supplemental figure 2).

T cell presence, indicated by CD3 positivity, was detected in the synovium of 12 out of the 50 RA-risk individuals and in all arthritis patients, in line with our previous data.^22^ DNA damage was observed in T cells, vimentin positive FLS and other synovial cells in RA-risk individuals, pre-RA and arthritis patients (figure 1C). The proportion of each cell type contributing to the total number of γH2AX positive cells within γH2AX positive synovium differed between the groups but was not statistically significant as the number of samples per group was limited (online supplemental figure 3). Across all study groups, the majority of γH2AX positivity was observed in FLSs and other synovial cells (figure 1C). Based on visual inspection, we noted more γH2AX positive cells in synovial biopsies positive for T cells. Indeed, the median percentage of γH2AX positive cells within the synovium of RA-risk individuals was statistically significantly increased (p value=0.0083) when CD3 positive T cells were also present (figure 1D). The presence of γH2AX positive cells alongside CD3 positive cells in the synovium of RA-risk individuals did not correlate with arthritis development, nor did the presence of γH2AX positive CD3 positive T cells (online supplemental figure 2).

Given that γH2AX presence was not related to arthritis development and the relatively small number of pre-RA synovial tissues, which were γH2AX positive, we next explored the cellular features of γH2AX positive synovium in the total RA-risk cohort. The severity of DNA damage in γH2AX positive synovium, indicated by the pixel intensity of γH2AX within nucleated cells, was shown to be statistically significantly increased in RA-risk synovium positive for CD3 T cells (p value=0.019) (figure 1E). When stratifying for cell type, we observed that the median pixel intensity of γH2AX per nucleated cell was statistically significantly (p value=0.007) increased particularly in vimentin positive FLS (median=37.83) from RA-risk synovium containing CD3 T cells compared with those lacking T cells (median=29.84) (figure 1F). This was not observed when comparing the median pixel intensity in other synovial tissue cells in the presence or absence of T cells (figure 1G) (p value=0.1469). Overall, these data suggest a persistent DNA damage in FLS in the presence of T cells. This persistent DNA damage is not present in other synovial cells while T cells are present.

### Increased DNA damage in RA-risk and RA FLS

**Figure 2:**
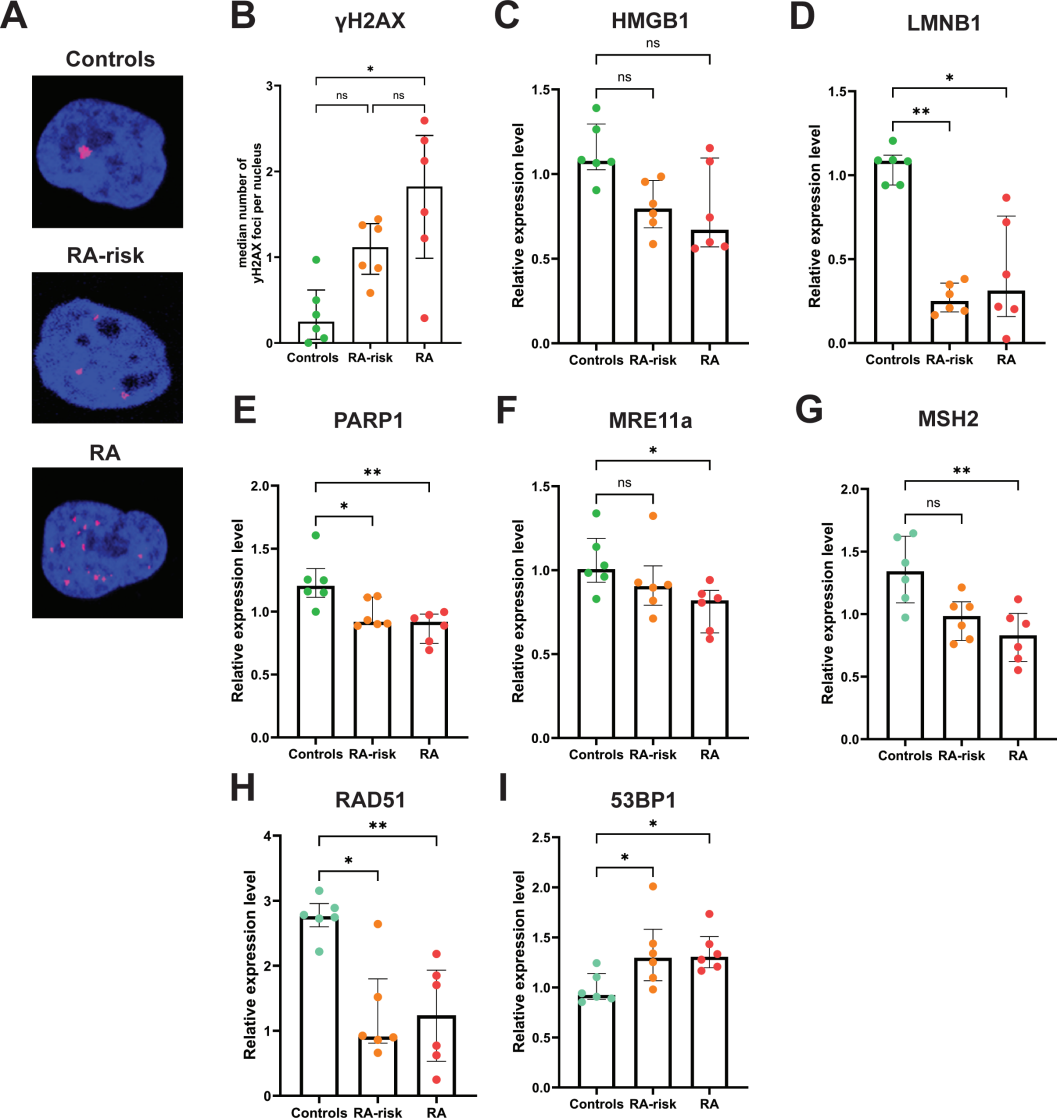
DNA damage in cultured synovial fibroblasts. Representative images (**A**) depicting γH2AX foci (pink) in nuclei of cultured control, RA-risk and RA FLS. Graph representing the median number of γH2AX foci per nucleus in untreated cultured FLS (**B**). The number of foci per cell was then determined by the number of γH2AX regions per DAPI region in approximately 50 cells per donor and condition. Graphs representing the relative expression levels of HMGB1 (**C**), LMNB1 (D), PARP1 (**E**), MRE11a (**F**), MSH2 (**G**), RAD51 (**H**) and 53BP1 (**I**) genes by qPCR. In all graphs, control (green circle), RA-risk (orange circle) and RA (red circle) untreated cultured FLS are depicted. Independent experiments of n=6 per group. Kruskal-Wallis T test followed by Dunn’s comparison test for multiple testing was used for all statistical analysis. *p value<0.05, **< 0.01. FLS, fibroblasts; ns, not significant; RA, rheumatoid arthritis.

We next determined whether the DNA damage observed in synovial tissue sections was maintained in ex vivo cultured FLS. FLS harvested and expanded from synovial tissue biopsies of RA-risk individuals, RA patients and age-matched controls were stained for γH2AX. The number of γH2AX positive foci per cell (figure 2A) indicates the number of double-stranded DNA breaks through recruitment and phosphorylation of H2AX. Thus, this is a proxy measurement of cellular DNA damage. Cultured FLS from RA-risk individuals and RA patients (p value=0.0063) showed a significant increase in the median number of foci per nucleus compared with control FLS (figure 2B). Overall, these findings show that the DNA damage observed in synovial tissue of RA-risk individuals and RA patients is maintained in cultured FLS.

To explore whether increased DNA damage in cultured FLS is coupled to molecular changes in DNA damage and repair-related genes, we performed qPCR on untreated cultured FLS from control, RA-risk and RA biopsies. The following genes were measured but did not differ significantly between control, RA-risk and RA FLS: FOXO4, BCL2L1, GLB1, EFNB1, CD38, NAMPT, SOD2, SIRT1, p53 (online supplemental figure 4), IL6, CDKN1A, CDKN2A, CDK4, MLH1, PMS2, BRCA2 and ERCC2. HMGB1 and LMNB1 are genes involved in genomic stability, telomere dynamics and chromatin organisation.^58 59^ HMGB1 (figure 2C) showed a trend towards a decrease in RA and RA-risk FLS when compared with controls. LMNB1 (figure 2D) showed a statistically significant decrease in RA-risk (p value=0.0028) and RA FLS (p value=0.0161) compared with controls. PARP1 is a chromatin-associated nuclear enzyme^60^ involved in regulating DNA repair mechanisms, which was also statistically significantly lower in RA-risk (p value=0.0299) and RA FLS (p value=0.0041) compared with controls (figure 2E). MRE11a is a gene involved in telomere maintenance and double-stranded DNA repair^61^ where it is directly recruited by γH2AX. MRE11a showed a statistically significant reduction in RA FLS (p value=0.0189) compared with control FLS. 53BP1 is a gene involved in non-homologous end joining, which was significantly increased in RA (p value=0.0221) and RA-risk (p value=0.0401) FLS compared with controls (figure 2I). RAD51 is a gene involved in homologous recombination as a repair mechanism for double-stranded DNA breaks, which unlike 53BP1 was significantly reduced in RA (p value=0.0059) and RA-risk (p value=0.0137) FLS compared with controls (figure 2H). MSH2, a gene involved in mismatch repair, was also significantly increased in RA (p value=0.0059) FLS compared with controls (figure 2G). This evidence for increased DNA damage coupled with molecular changes in key DNA damage repair genes led us to explore whether there was an intrinsic loss in DNA repair capacity in cultured FLS.

### Impaired DNA repair in FLS from RA and RA-risk individuals

**Figure 3:**
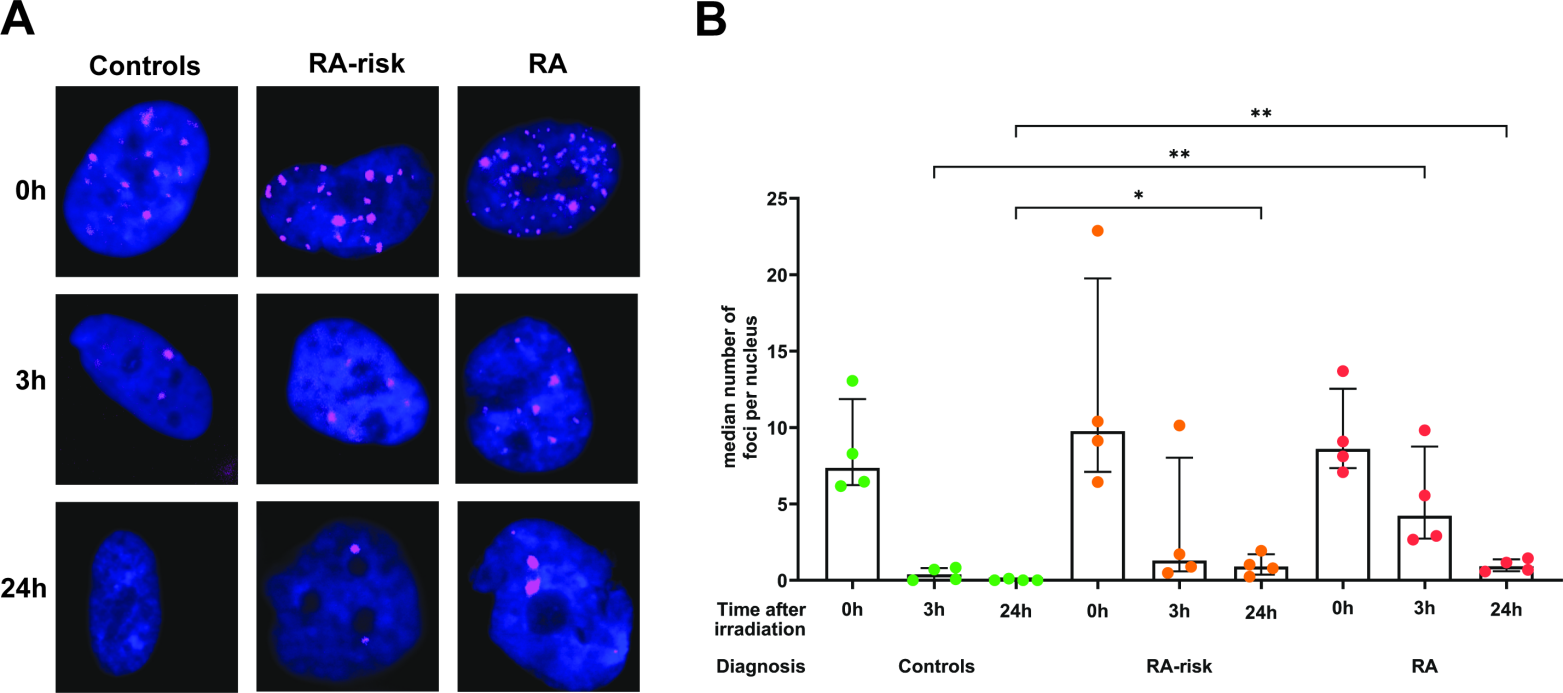
DNA repair capacity in cultured synovial fibroblasts. Representative images (**A**) depicting γH2AX foci (pink) in nuclei of irradiated cultured control, RA-risk and RA FLS at 0 hours, 3 hours and 24 hours post irradiation. The number of foci per cell was then determined by the number of γH2AX regions per DAPI region in approximately 50 cells per donor and condition. In all graphs, control (green circle), RA-risk (orange circle) and RA (red circle) irradiated cultured FLS are depicted. Independent experiments of n=4 per group. A two-way ANOVA with a Sidak’s multiple comparison test was used for all statistical analysis. *p value<0.05, **< 0.01, only statistically significant comparisons are shown. FLS, fibroblasts; RA, rheumatoid arthritis.

To determine whether the increased DNA damage observed in cultured RA and RA-risk FLS was allied with an intrinsic defect in their DNA repair mechanisms, we induced DNA damage by irradiation and followed the cellular DNA damage repair response. Following irradiation, cultured FLS from RA, RA-risk and controls were stained for γH2AX at specified time points to track their repair capacity (figure 3). In all three study groups, irradiation induced significant DNA damage, which was at least partially repaired after 24 hours (figure 3A). The median number of foci per nucleus immediately post-irradiation was not statistically significantly different between control, RA-risk and RA FLS (figure 3B). As expected, control FLS were able to fully repair the induced DNA damage within 24 hours as indicated by the lack of γH2AX foci. In both RA-risk and RA cultured FLS, positive γH2AX foci were still detected in the nucleus, 24 hours post-irradiation with medians of 0.9 and 1.9 respectively, indicative of incomplete DNA repair (figure 3B). The median number of foci per nucleus, 3 hours post-irradiation, was on average higher in RA-cultured FLS (median=5.5) compared with RA-risk (median=1.1) and control FLS (median=0.3). This may suggest there is a slower rate of DNA repair in the RA FLS. Altogether, these data suggest that RA and RA-risk FLS have a reduced capacity to repair DNA damage.

### Dasatinib partially restores DNA repair capacity of FLS from RA and RA-risk individuals

**Figure 4:**
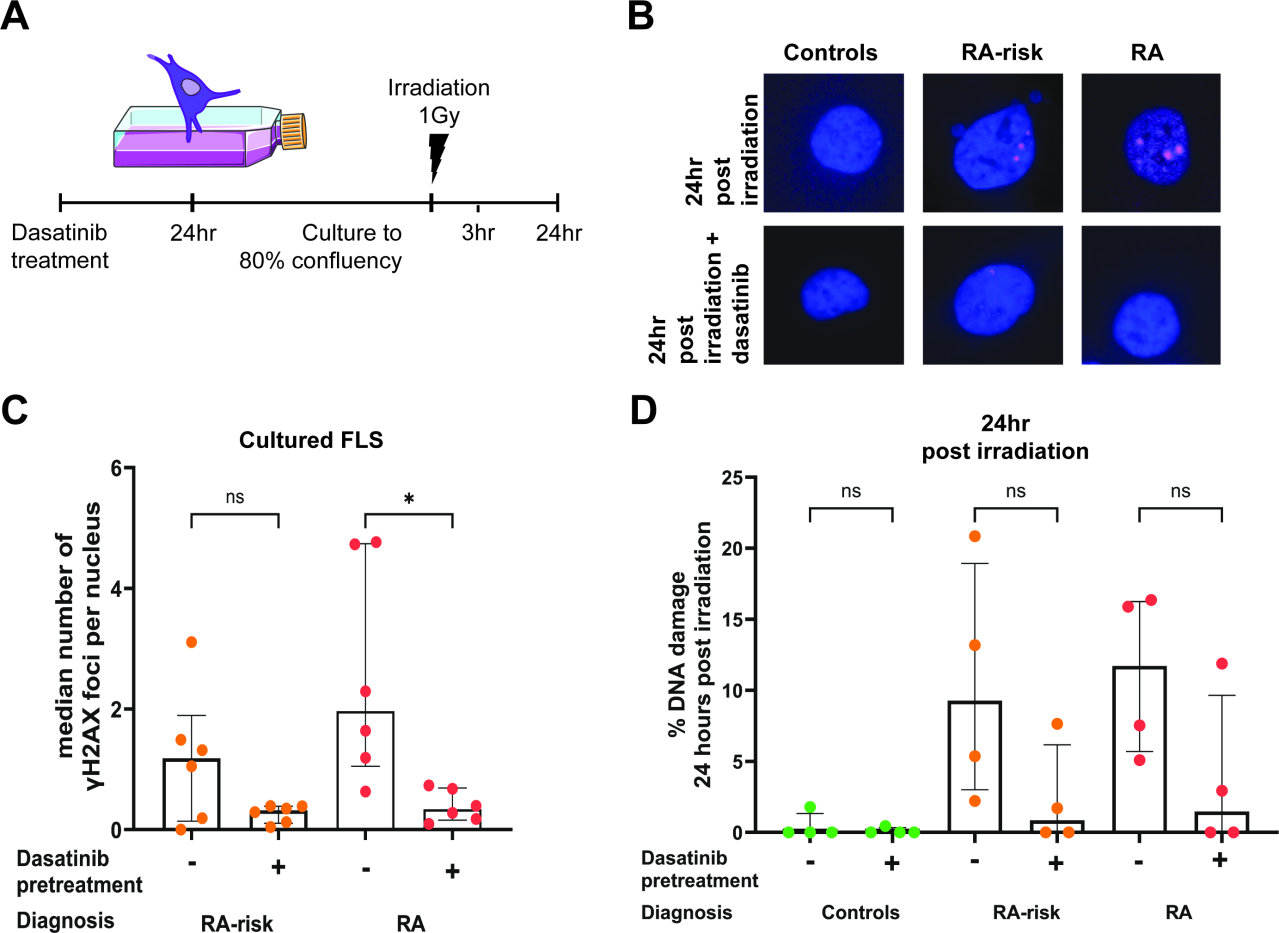
Dasatinib treatment partially restored DNA damage and repair in cultured synovial fibroblasts. Schematic (**A**) depicting the culture regime for pretreatment with dasatinib before irradiation of cultured FLS. Representative images (**B**) depicting γH2AX foci (pink) in nuclei in cultured FLS following 24 hours irradiation and dasatinib treatment. The number of foci per cell was then determined by the number of γH2AX regions per DAPI region in approximately 50 cells per donor and condition. Graph representing the median number of γH2AX foci per nucleus in dasatinib treated and untreated cultured FLS (**C**). Graph representing the percentage of DNA damage remaining in dasatinib treated and untreated cultured FLS 24 hours after irradiation to induce DNA damage (**D**). In all graphs, control (green circle), RA-risk (orange circle) and RA (red circle) untreated cultured FLS are depicted. Independent experiments of n=6 per group for untreated and n=4 per group for irradiated FLS. Kruskal-Wallis T test followed by Dunn’s comparison test for multiple testing was used for all statistical analysis. *p value<0.05, **< 0.01, FLS, fibroblasts; ns, not significant; RA, rheumatoid arthritis.

To determine whether this reduced DNA repair capacity could be restored, we treated cultured FLS with dasatinib, a tyrosine kinase inhibitor that primarily targets senescent cells.^62^ Treatment of cultured FLS with dasatinib reduced the DNA damage observed at baseline (figure 4C). This pretreatment eliminated the previously observed increased DNA damage in RA and RA-risk FLS. Dasatinib treatment of RA FLS (p value=0.0146) induced a statistically significant decrease in the median number of foci per nucleus. To explore the effect of dasatinib pretreatment on DNA repair capacity, cultured FLS following dasatinib treatment were irradiated in the same manner as described previously (figure 4A). We did not observe any discernible differences in γH2AX foci immediately or 3 hours post irradiation between controls, RA-risk and RA FLS (online supplemental figure 3). We did observe a trend towards a decrease in γH2AX foci at 24 hours post-irradiation in RA and RA-risk FLS when FLS had been treated with dasatinib (figure 4B,D). This evidence suggests that treatment of FLS with dasatinib to eliminate senescent cells, reduced DNA damage and partially restored the overall DNA repair capacity of cultured FLS.

## Discussion

Cellular and molecular hallmarks of premature ageing have been implicated in RA pathogenesis, including telomere shortening,^26^,^28^ cellular senescence and genomic instability.^35^ FLS^63^ and T cells^36^ have both been shown to exhibit senescent phenotypes, which accumulate in RA. Despite this knowledge, it is unclear whether senescent cells play a pertinent role in initiating a chronic inflammatory environment or are a consequence of it. This study is the first to explore genomic instability in the form of DNA damage in synovium during the preclinical phase of RA. This study builds on previous work exploring the cellular^23 64^ and molecular changes^24^ that occur in the synovium preceding arthritis development.^5 22^ Our study is the first, to our knowledge, to show persistent DNA damage in preclinical synovium from RA-risk individuals. We highlight that FLS from RA-risk individuals and RA patients have increased DNA damage and a reduced capacity to repair DNA, which can be partially restored on senolytic treatment to eliminate senescent cells.

DNA damage arises in cells during homeostasis due to endogenous triggers such as reactive oxygen species and replication errors, which is continuously repaired in healthy tissues.^65 66^ DNA damage is measured in tissues to determine the genomic stability of cells and is often used in oncology to identify precancerous cells.^67^ H2AX is phosphorylated at Serine 139^44^ and forms foci indicating double-stranded DNA breaks and allowing for the recruitment of further DNA repair machinery. This γH2AX can be detected by immunofluorescence as a measure of DNA damage. Accordingly, γH2AX staining in synovial tissue of RA-risk individuals indicates the presence of double-stranded DNA breaks. While γH2AX marks both physiological and pathological DNA damage, functional experiments in ex-vivo cultured FLS show that irradiation-induced DNA damage is repaired less efficiently in FLS from RA-risk and RA patients compared with controls. Together, these findings suggest that the observed γH2AX staining in synovial tissue reflects, at least in part, a delayed or impaired DNA damage response in FLS rather than solely random or transient DNA lesions.

The presence of γH2AX in the synovium of both RA patients and individuals at risk, prior to overt inflammation, and its elevation in FLS adjacent to T cells, suggests that FLS–T cell interactions may contribute to an altered synovial microenvironment. Such alterations in the environment may consequently predispose it to chronic inflammation. Previous studies show that RA synovium is characterised by hyperplasia due to FLS expansion, immune cell infiltrate and a dysfunctional cellular environment.^10 15 16 68^ In particular, RA FLS have recently been shown to have increased p16 and beta galactosidase, which are associated with cellular senescence.^63^ This senescent phenotype was increased in RA FLS compared with OA and healthy FLS in younger individuals. This finding highlights how senescence is also a product of normal ageing in healthy FLS but happens prematurely in the context of RA FLS contributing to an aberrant synovial environment. Our previous work exploring RA-risk synovium highlighted no overt synovial inflammation in this phase and only a subtle increase in T cells in pre-RA synovium.^22^ Here, we confirm the modest CD3 T cell infiltration in only a proportion (12/50) of RA-risk synovium. This CD3 presence did not directly correlate with the presence of γH2AX, and the presence of CD3 T cells was not always accompanied by DNA damage. Nevertheless, DNA damage in FLS was higher in the presence of synovial T cells. The presence of DNA damage in RA-risk synovium suggests that DNA damage precedes chronic synovial inflammation. DNA damage was not present in all RA-risk individuals, which suggests there may be a subset of individuals that are predisposed to persistent DNA damage likely due to a combination of environmental and genetic factors, which have hitherto not been revealed. The location of DNA damage and the cell types affected did not differ significantly between RA-risk and arthritis synovium, suggesting there is not a discernible shift in the cells exhibiting DNA damage between the preclinical risk phase and established disease, and the mere presence of DNA damage was not predictive of arthritis development.

Increased DNA damage was observed when comparing RA-risk synovium with and without T cell infiltrate. The presence of T cells is found in healthy synovium at low levels in the perivascular region^69 70^ and has previously been shown to modestly correlate with synovial inflammation.^64^ In our cohort, when T cells were present alongside DNA damage, there was increased DNA damage specifically in FLS. This may indicate a more senescent environment with low-grade inflammation; however, further research is required to determine whether the DNA damage observed in these cells is present before synovial infiltration or the result of an aberrant microenvironment. Moreover, currently, it is unknown whether T cells present within the synovium of RA-risk individuals are resident cells or have infiltrated the synovium as an initiating event steering arthritis development. Furthermore, the severity of the DNA damage, indicated by increased γH2AX intensity, is increased in synovium with T cell presence. This may suggest that the altered microenvironment induced by T cells makes FLS more susceptible to DNA damage, or that the altered synovial microenvironment attracts T cells. The association between DNA damage in FLS and the presence of T cells suggests a link between DNA damage and immune cell infiltrate, which warrants further investigation.

Several studies^71^,^74^ have highlighted how cultured FLS mimic the synovial environment from which they originate. Indeed, the observed DNA damage detected in RA-risk synovium is maintained in cultured FLS. Cultured RA-risk and RA FLS had increased γH2AX foci compared with control FLS accompanied by a reduced capacity to repair induced DNA damage and aberrant expression of DNA repair genes. This evidence supports the idea that FLS are already dysfunctional in the preclinical phase of disease^24 54^ and key drivers of disease pathogenesis. Our observations are in line with increasing knowledge that FLS contribute to chronic inflammation by creating a pro-survival environment for immune cells in the joint following a loss in immunomodulatory function in early RA.^75 76^ RA FLS can promote endothelial cell recruitment of lymphocytes in vitro, which did not occur when tested using skin FLSs from the same RA patients.^77 78^ This suggests that this property is intrinsic to the FLS residing directly in the synovial environment rather than an overarching function of tissue resident FLS. In turn, the specificity of this observation only in FLS may contribute to why there are clinical manifestations only within the joint.

Furthermore, FLS from non-inflamed joints or resolving arthritis were immunosuppressive in culture compared with those from early RA and established RA patients.^75^ The accumulation of epigenetic modifications in FLS^79^,^81^ likely drives disease progression from early to established RA, but this continuum has yet to be determined for the preclinical phase. The lack of full DNA repair observed in RA-risk and RA FLS is likely due to the presence of senescent FLS, which is reflected by the positive effect of dasatinib pretreatment. Dasatinib is part of a group of senolytic drugs, which target senescent cells.^62 82^ It is likely that the presence of these senescent cells, be it FLS or otherwise, in RA and RA-risk synovium promotes chronic inflammation through the release of senescence-associated secretory cytokines and an incapacity to repair DNA damage creating genomic instability. Targeting of senescent cells^83^ and particularly FLS in RA is currently being tested in a clinical trial^84^ and may provide therapeutic benefit. However, the efficacy of this therapeutic targeting in a preclinical phase is currently unknown and may prove challenging. Nevertheless, targeting pathways that modulate DNA damage responses might represent a novel strategy for preventing or delaying the onset of RA in at-risk individuals.

Overall, this study identifies DNA damage in the synovium prior to RA onset, raising the possibility of impaired DNA repair as an early feature of disease. However, the small cohort size and limited number of pre-RA samples restrict the strength of these conclusions, and validation in larger, prospective studies will be essential. Careful investigation of the genetic and environmental factors contributing to DNA damage in RA-risk individuals will be necessary to clarify its role in synovitis and disease progression.

## Supplementary material

10.1136/rmdopen-2025-005774 online supplemental file 1

## Data Availability

All data relevant to the study are included in the article or uploaded as supplementary information.
