# Supplementary material for: RA-risk synovium exhibits DNA damage coupled with impaired DNA repair in fibroblasts
Source: RMD Open. 2026 Jan 22;12(1):e005774. doi: 10.1136/rmdopen-2025-005774 (PMC12829405; doi:10.1136/rmdopen-2025-005774)
Supplement: online supplemental file 1 [file rmdopen-12-1-s001.docx]

**Supplementary Figure 1: Immunofluorescence staining of synovial tissue sections**

**Supplementary Figure 2: Correlations of synovial tissue populations and clinical parameters**

**Supplementary Figure 3: Proportions of synovial cell subsets in γH2AX positive cells**

**Supplementary Figure 4: p53 gene expression in synovial fibroblasts**

**Supplementary Figure 5: DNA repair capacity of cultured FLS following dasatinib treatment**


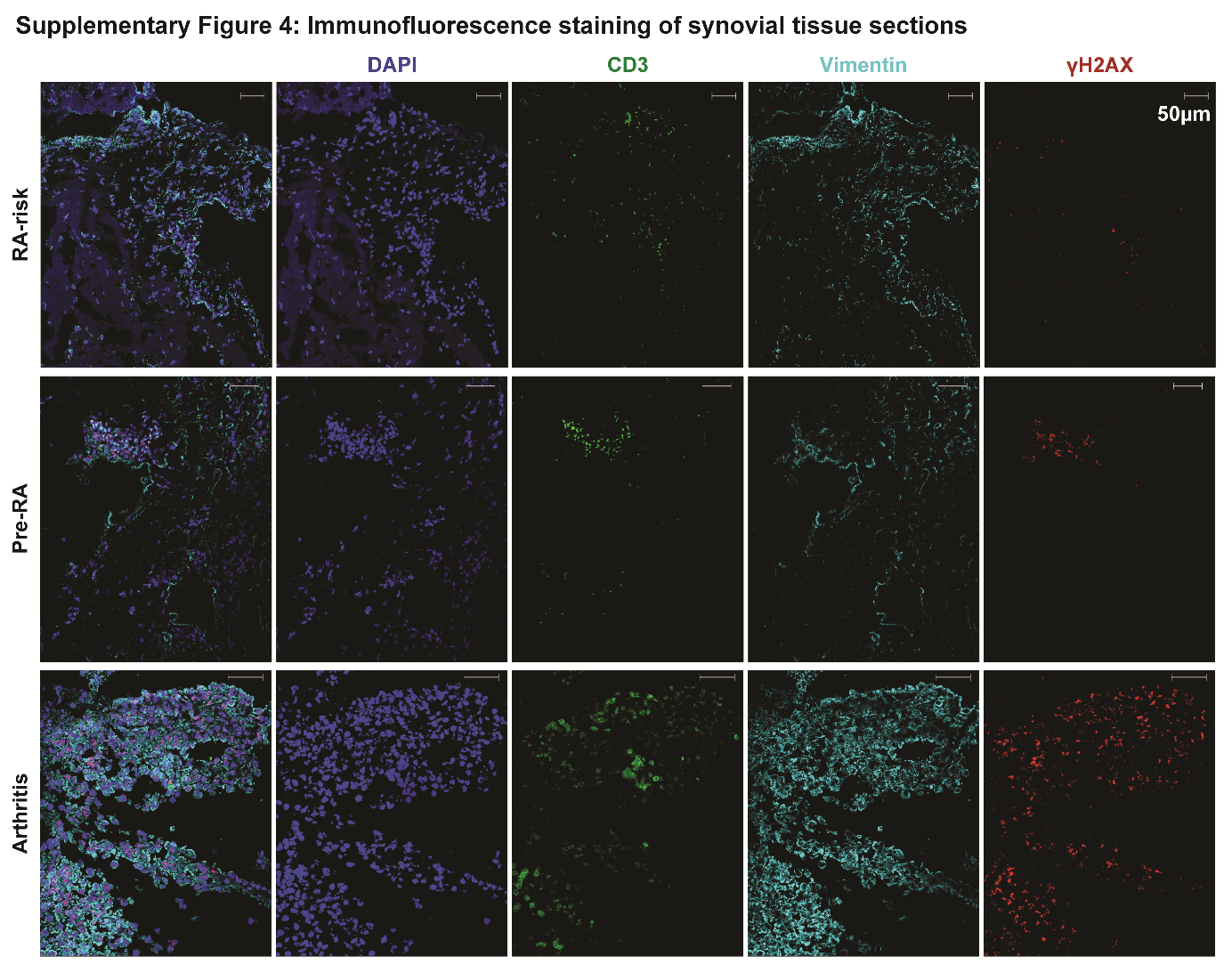


**Supplementary Figure 1: Immunofluorescence staining of synovial tissue sections**

Representative images of synovial tissue staining for nuclei (DAPI, blue), T cells (CD3,green), fibroblasts (vimentin, cyan) and DNA damage (γH2AX,red) from RA-risk, pre-RA and arthritis patients.


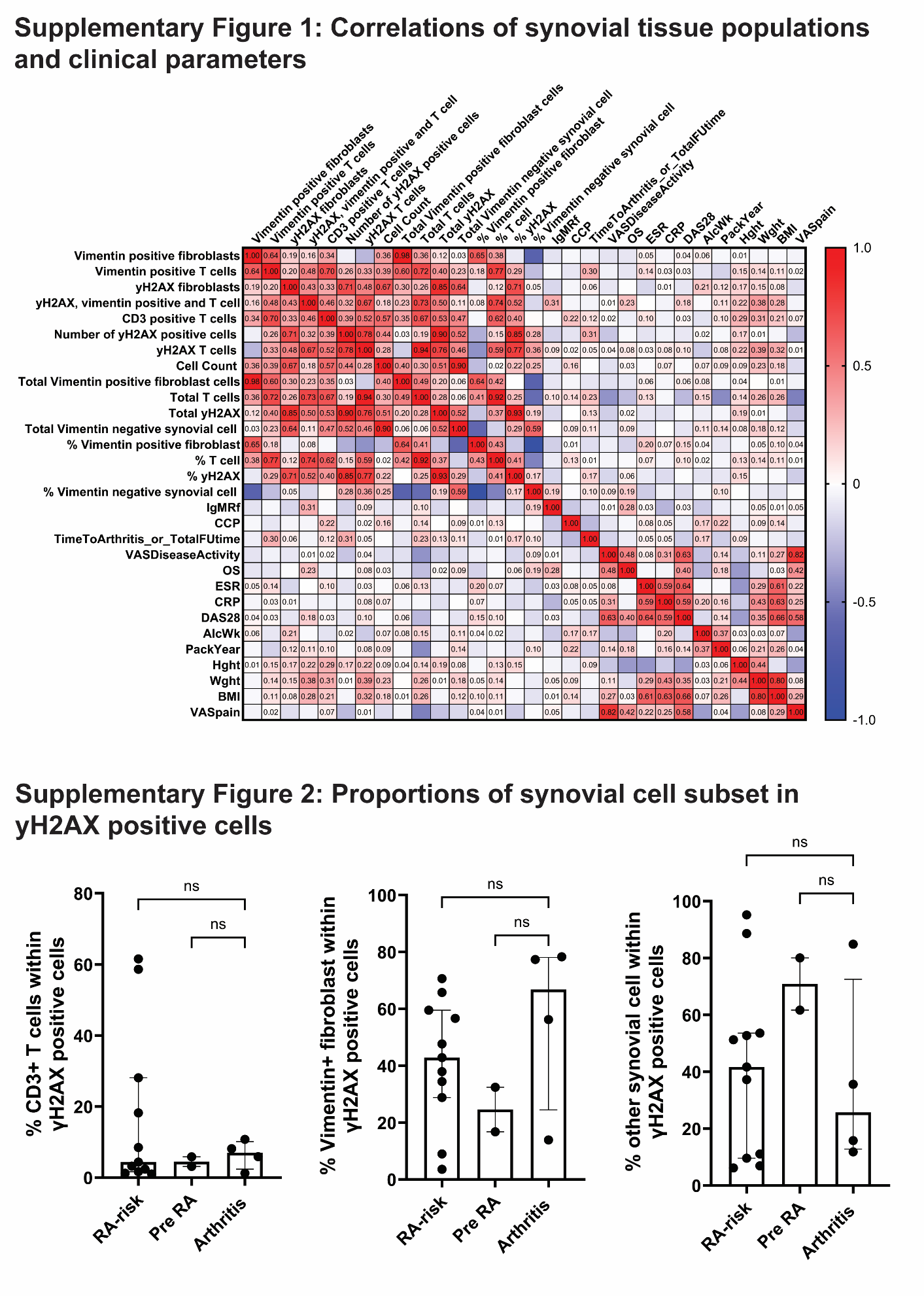


**Supplementary Figure 2: Correlations of synovial tissue populations and clinical parameters**

Heatmap depicting Spearman’s correlation coefficients for categorized synovial tissue populations from QuPath image analysis of synovial tissue cohort and known clinical parameters. Red and blue shaded squares present positive and negative correlation coefficients respectively. IgM-RF, IgM rheumatoid factor titre, CCP, citrullinated peptide titre, OS, morning stiffness, VAS, visual analogue scale, ESR, erythrocyte sedimentation rate, CRP, C reactive protein, DAS28, disease activity scale 28, AlcWk, alcohol units per week, PackYear, cigarette packets per year, BMI, body mass index.


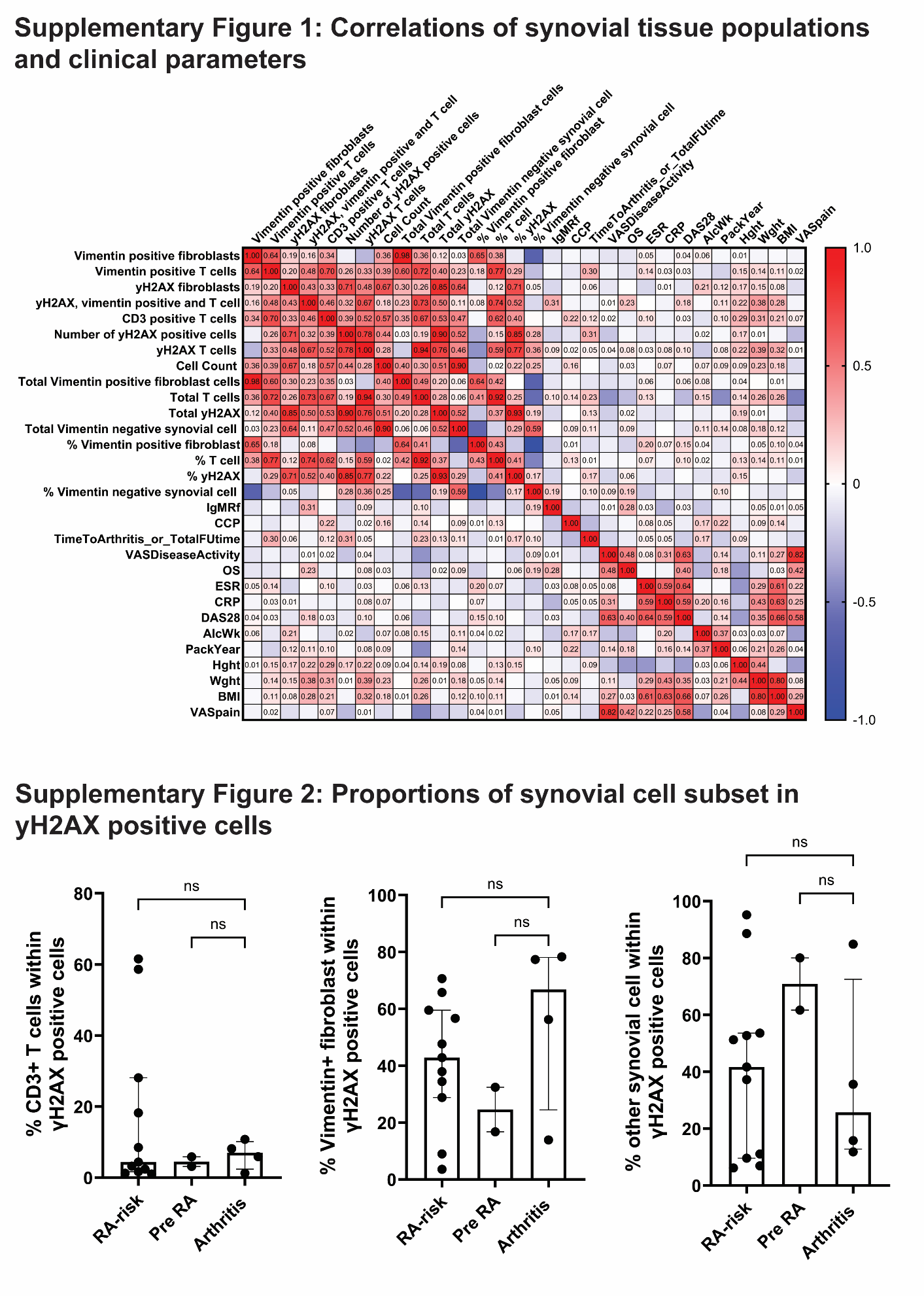


**Supplementary Figure 3: Proportions of synovial cell subsets in γH2AX positive cells**

Bar charts presenting the percentage of CD3 positive T cells, vimentin positive fibroblasts and other synovial cells within the total population of γH2AX positive cells in RA-risk, pre-RA and arthritis synovial tissue sections. RA-risk (n = 11), pre-RA (n = 2) and arthritis (n = 4) sections. Kruskal Wallis T test performed for statistical analysis.

**Supplementary Figure 4: p53 gene expression in synovial fibroblasts.**

Bar chart presenting the relative expression of p53 gene by quantitative PCR in synovial fibroblasts from controls, RA-risk and RA patients (all n = 6).


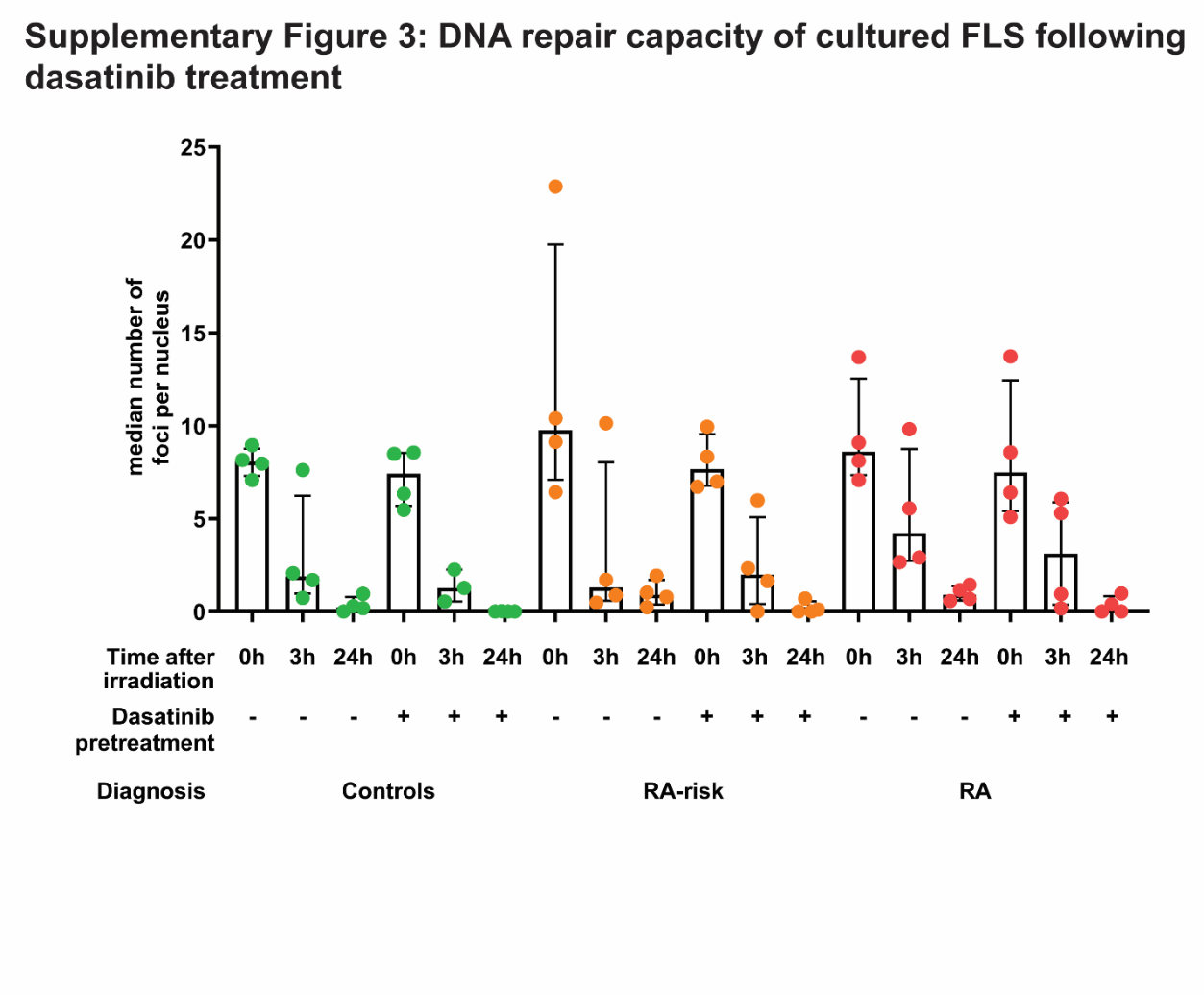


**Supplementary Figure 5: DNA repair capacity of cultured FLS following dasatinib treatment**

Bar chart depicting the median number of foci per cell following irradiation +/- dasatinib treatment in RA (red dots), RA-risk (orange dots)and control (green dots) FLS. γH2AX positivity was measured immediately, 3 hours and 24 hours post irradiation in four donors. FLS were used in passages 3-5.
